# Fulvestrant: pharmacokinetics and pharmacology

**DOI:** 10.1038/sj.bjc.6601630

**Published:** 2004-03-05

**Authors:** J F R Robertson, M Harrison

**Affiliations:** 1 Unit of Surgery, City Hospital, Nottingham NG5 1PB, UK; 2 AstraZeneca Pharmaceuticals, Macclesfield, Cheshire, UK

**Keywords:** fulvestrant, ‘Faslodex’, oestrogen receptor, pharmacokinetics, metabolism

## Abstract

Fulvestrant is a new type of oestrogen receptor (ER) antagonist with no agonist activity and a novel pharmacological profile. Fulvestrant has been shown to significantly reduce cellular levels of the ER and progesterone receptor in both preclinical studies and in clinical trials of postmenopausal women with primary breast cancer. This paper reviews the pharmacokinetics and metabolism of fulvestrant, which support the rationale for drug delivery as a single, once-monthly intramuscular injection, and show that this agent has minimal potential to be the subject, or cause, of significant cytochrome *p*450-mediated drug interactions.

Fulvestrant (‘Faslodex’) is an oestrogen receptor (ER) antagonist with no agonist effects and a novel pharmacological profile, downregulating cellular levels of both the ER and progesterone receptor (PgR) (Howell *et al*, 2000; Robertson *et al*, 2001). Fulvestrant competes with oestrogen for binding to the ER. In order to achieve effective inhibition of oestrogen-controlled proliferation, downregulation of the ER must be sustained over time. Therefore, in contrast to some other antitumour agents (such as cytotoxic chemotherapies, which are administered in cyclic regimens) sustained exposure to fulvestrant via chronic administration is required for activity. Oral delivery was explored in animals and man using a range of formulations; however, the low level of bioavailability and presystemic metabolism meant that this was not an appropriate route of administration (Harrison *et al*, 2003). Therefore, a long-acting, intramuscular (i.m.) formulation of fulvestrant was developed that gives adequate bioavailability and allows controlled release of the drug. This paper will review fulvestrant pharmacokinetic and metabolism data that have been obtained from preclinical studies and from trials in healthy volunteers and in postmenopausal women with breast cancer.

## PHARMACOKINETICS OF FULVESTRANT

### Single-dose intravenous studies

An intravenous (i.v.) formulation of fulvestrant was developed for use in pharmacokinetic investigations since plasma concentrations of fulvestrant administered by the i.m. route are determined by the rate of release of the drug from the injection site and, therefore, do not accurately reflect the elimination kinetics of the compound. A short-acting i.m. formulation was used to mimic steady-state plasma concentrations obtained using long-acting fulvestrant, and to overcome the difficulties of obtaining pharmacokinetic data with the long-acting formulation.

The pharmacokinetics of a number of doses of i.v. fulvestrant have been determined in healthy men and postmenopausal women. Following i.v. administration, fulvestrant plasma concentrations increased rapidly, reaching an approximate steady state by the end of the 1-h infusion. Plasma concentrations declined in a triexponential manner, falling very rapidly with an approximate five-fold reduction within 30 min (Harrison *et al*, 2003). Over several single-dose i.v. studies, the terminal elimination half-life (*t*_1/2_) of i.v. fulvestrant ranged from 13.5 to 18.5 h. Fulvestrant was subject to extensive and rapid distribution, with estimates of the volume of distribution (*V*_ss_) at the steady state ranging from 3.0 to 5.3 l kg^−1^. Clearance of the compound from the plasma was high, with mean values between 9.3 and 14.3 ml min kg^−1^, which is similar to hepatic plasma flow (nominally 10.5 ml min kg^−1^), and suggests that the compound is cleared by the liver and possibly by extrahepatic metabolism. Pharmacokinetic modelling showed that the plasma concentration data fitted a three-compartment infusion model (Harrison *et al*, 2003).

### Single-dose i.m. studies using the short- and long-acting fulvestrant formulations

Single-dose pharmacokinetic studies using various doses (2, 6, 18 and 36 mg) of the short-acting i.m. fulvestrant formulation in healthy postmenopausal female volunteers indicated slow absorption with maximum plasma concentrations (*C*_max_) being achieved at 12–24 h postinjection. In contrast, following single i.m. injections of long-acting fulvestrant at doses of up to 250 mg in various trials, the time to *C*_max_ (*t*_max_) varied between 2 and 19 days, indicating prolonged release of fulvestrant from the injection site. After *C*_max_, plasma concentrations of fulvestrant declined slowly and it was possible to define plasma profiles over at least 28±3 days (i.e. the intended dosing period) in most subjects after a single dose, and profiles were approximately log-linear over this time scale (Harrison *et al*, 2003). Pharmacokinetic parameters following a single dose of long-acting i.m. fulvestrant in healthy postmenopausal female volunteers and in postmenopausal women with advanced breast cancer are shown in Table 1

**Table 1:** Pharmacokinetic parameters following a single dose of long-acting i.m. fulvestrant 250 mg in healthy female postmenopausal volunteers (AstraZeneca, data on file) and in postmenopausal women with advanced breast cancer (Trial 0020, Robertson *et al*, 2003a)

	**Healthy volunteers**	**Breast cancer patients (study 0020)**
AUC_(0–28)_ (ng ml^−1^) gmean (CV, %)	176 (34.5) (*n*=10)	148 (45.3) (*n*=13)
*C*_max_ (ng ml^−1^) gmean (CV, %)	11.4 (44.8) (*n*=10)	8.20 (63.8) (*n*=16)
*t*_max_ (h) median	144.0 (*n*=10)	167.3 (*n*=15)
*C*_min_ (ng ml^−1^) gmean (CV, %)	2.6 (25.2) (*n*=10)	2.62 (33.4) (*n*=14)

gmean=geometric mean; SD=standard deviation; CV=coefficient of variation; AUC_(0–28)_= area under the plasma concentration–time curve from 0 to 28 days; *C*_max_=maximum plasma concentration; *t*_max_=time to *C*_max_; *C*_min_=minimum plasma concentration.

. The pharmacokinetic behaviour of fulvestrant after a single long-acting 250 mg i.m. dose appears to be similar in these two populations.

**Figure 1:**
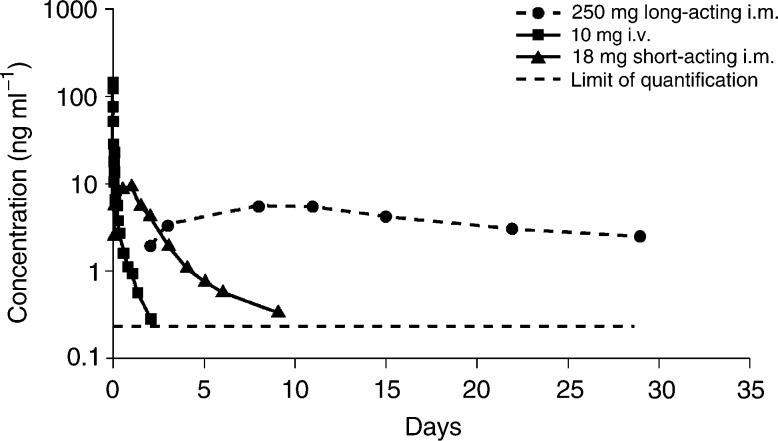
Mean plasma concentrations of fulvestrant following i.v. and i.m. (short-acting or long-acting) administration.

Pharmacokinetic modelling of pooled data from breast cancer patients given a single 250 mg dose of long-acting i.m. fulvestrant suggest that the plasma concentration–time data fit a two-compartment model. The different sampling frequency used to gather data in the i.v. study enables a more complex three-compartment model to be assigned. The apparent *t*_1/2_ observed in these studies (40 days) was approximately 40 times longer than that of an i.v. injection of fulvestrant (AstraZeneca, data on file). Comparisons of fulvestrant plasma profiles obtained following i.v. or i.m. administration show the marked effects of the i.m. formulations (short-acting or long-acting) on the release of fulvestrant into the circulation compared with i.v. administration (Figure 1

). In particular, the difference between the two i.m. formulations clearly demonstrates the prolonged release characteristics of the long-acting formulation.

An analysis of the single-dose (50, 125, 250 mg) pharmacokinetics from a trial of long-acting i.m. fulvestrant in postmenopausal women with previously untreated primary breast cancer showed that *C*_max_ and area under the plasma concentration–time curve from zero to 28 days (AUC_(0–28)_) appeared to increase in a dose-related manner (Robertson *et al*, 2001). Formal statistical analysis of covariance indicated that exposure was approximately proportional to the dose (proportionality coefficient: 0.88; 95% confidence interval (CI) 0.74–1.03; *P*=0.1112).

### Multiple-dose i.m. studies using the long-acting formulation of fulvestrant

Fulvestrant has been licensed in the USA since April 2002. Here, nursing guidelines have previously suggested that, for adults, i.m. injections into large muscles such as the gluteus medias should not usually exceed 4 ml (Beyea and Nicoll, 1996). Therefore, when a US-based Phase III study was initiated, it was decided to use 2 × 2.5 ml i.m. fulvestrant injections, one into each buttock, rather than a single 5 ml i.m. fulvestrant injection (Howell *et al*, 2002). However, the 2 × 2.5 ml injections were sufficiently well tolerated (with a similar incidence of injection-site reactions observed for placebo and fulvestrant), that most US institutions now prefer to administer fulvestrant as a single 5 ml monthly injection, thereby reducing the number of injections given to the patient (AstraZeneca, data on file). Similar pharmacokinetic properties of the 1 × 5 ml and 2 × 2.5 ml fulvestrant regimens have been demonstrated in a study of postmenopausal women with advanced breast cancer. No significant differences in the AUC, plasma concentration at 28 days (*C*_min_), and ratio of geometric means of AUC_0–28_ (1.01; 95% CI 0.68–1.51; *P*=0.94) were observed between the two regimens (Robertson and Harrison, 2003). When i.m. fulvestrant 250 mg was given monthly until disease progression, fulvestrant plasma concentration profiles reached a steady state after 3–6 doses. The predicted steady-state plasma fulvestrant exposures (AUC_ss_) were very similar for the two dose regimens (1 × 5 ml: 336 ng day ml^−1^; 2 × 2.5 ml: 294 ng day ml^−1^). Both regimens were equally effective in maintaining plasma levels for at least 30 months (Robertson and Harrison, 2003). With repeated administration, there was no evidence of a change in the pharmacokinetic behaviour of fulvestrant compared with that observed following the first injection, and Phase III trial data have since supported this observation (Robertson *et al*, 2003). Multiple-dose pharmacokinetics were modelled, and showed that blood levels of fulvestrant were maintained within a narrow range throughout the assessment period, thus confirming the linearity of fulvestrant pharmacokinetics during repeated administration over a prolonged period.

In a small study where 19 postmenopausal patients with tamoxifen-resistant advanced breast cancer were given fulvestrant 250 mg (1 × 5 ml i.m. injection, monthly), the *C*_max_ for fulvestrant during the first month was 10.5 ng ml^−1^ and was 12.6 ng ml^−1^ during the sixth month. Similarly, the AUC rose from 140.5 ng day ml^−1^ in the first month to 206.8 ng day ml^−1^ in the sixth month, suggesting some drug accumulation (Howell *et al*, 1996). Two- to three-fold accumulation of fulvestrant with continued dosing has also been observed in several other studies using long-acting i.m. fulvestrant (DeFriend *et al*, 1994; Robertson and Harrison, 2001). For example, in one of these studies, the mean trough concentrations of i.m. fulvestrant were found to be 6.1 ng ml^−1^ after 6 months of fulvestrant 250 mg treatment compared with 2.8 ng ml^−1^ after the first month (Robertson and Harrison, 2001). In summary, the long-acting 250 mg i.m. formulation (administered every 28±3 days) is convenient for the patient, allowing sustained exposure to fulvestrant with plasma levels maintained over the predicted biological threshold for at least 28 days.

## METABOLISM OF FULVESTRANT

*In vitro* studies have indicated that fulvestrant is extensively metabolised. The metabolism of [^14^C]-fulvestrant following i.m. administration was initially investigated in animal studies (rats and dogs), which showed that fulvestrant was highly metabolised and mostly excreted in faeces (indicative of biliary metabolism). Little renal excretion was evident. The metabolite profiles were similar in rats and dogs, differing only in the relative proportions of individual components. The major excretory metabolites in these species were fulvestrant and its 17-ketone and/or sulphone analogues (rat: 15–20%; dog: 61%) together with material corresponding to sulphate conjugates (up to 16%). These results were consistent with earlier *in vitro* data (AstraZeneca, data on file).

The metabolism of both i.v. and i.m. [^14^C]-fulvestrant has been investigated in man. The i.v. trial was an open study in which four male and four postmenopausal female volunteers each received 10 mg [^14^C]-fulvestrant, as a 1-h i.v. infusion. Following administration, distribution of the drug was rapid, with plasma levels declining soon after the infusion, and, by 2 h, postinfusion geometric mean (gmean) levels of only 15.6 and 12.8 ng ml^−1^ were detected in male and female volunteers, respectively. At the end of the 1-h infusion, [^14^C]-fulvestrant accounted for approximately 80% of the total plasma radioactivity, declining to about 30% after 2 h. This suggests that fulvestrant is quickly metabolised when administered intravenously.

The i.m. study was also an open trial, including four male and three postmenopausal female volunteers, each of whom received a single i.m. 18 mg dose of short-acting [^14^C]-fulvestrant, in order to minimise the duration of exposure of the volunteers to the radioactive label. Following injection, slow absorption resulted in low levels of [^14^C]-fulvestrant being maintained for several hours (gmean of 14.6 ng ml^−1^ at 8 h in males and 13.3 ng ml^−1^ at 24 h in females). At 1 h postinjection, [^14^C]-fulvestrant accounted for approximately 90% of the total plasma radioactivity, thereafter decreasing to about 50% and declining further after 24 h. The majority of fulvestrant and/or its metabolites were associated with the plasma rather than the cellular components of the blood (typically 30–70% higher values for plasma compared with whole blood) in both the i.v. and i.m. studies (Harrison *et al*, 2003).

**Figure 2:**
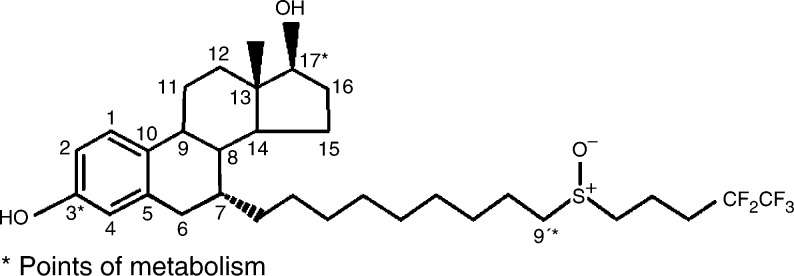
Structure of fulvestrant and points of metabolism.

As seen in animals, the major route of excretion was via the faeces (approximately 80 and 90% in the i.v. and i.m. trials, respectively), with less than 1% being excreted in the urine. In general, the faecal metabolite profiles of [^14^C]-fulvestrant were similar after i.v. or i.m. delivery, although the proportion of each type of metabolite differed depending on the route of administration. Fulvestrant was converted at the 3- and 17-positions of the steroid nucleus to form ketone, sulphate and glucuronide metabolites, and at the 9-position to form sulphone metabolites (Harrison *et al*, 2003) (Figure 2

). These metabolites have been synthesised and tested for pharmacological activity *in vitro*. These studies have shown that all the putative metabolites possess no oestrogenic activity and only the 17-keto compound demonstrated a level of antioestrogenic activity of the same order of magnitude, but 4.5-fold less than that of fulvestrant.

## POTENTIAL FOR CYP-MEDIATED DRUG INTERACTIONS

Cytochrome *p*450 (*p*450 or CYP) is an enzyme responsible for the metabolism of a wide variety of drugs (Dresser *et al*, 2000) including many anticancer agents (Kivisto *et al*, 1995, 1998; Crewe *et al*, 1997). Therefore, it is important to investigate the potential for CYP interactions to occur with any new drugs in development. In *in vitro* studies, human liver microsomal protein was incubated with selected CYP substrates in the presence of a range of concentrations of fulvestrant (up to 2 *μ*g ml^−1^). Although concentration-related inhibition of some of the enzymes (CYP1A2, 2C9 and 3A4) was apparent, these effects were minimal, with less than 20% inhibition occurring at the highest concentration tested (AstraZeneca, data on file). Fulvestrant did not inhibit the CYP 2C19 and 2D6 isoforms.

The marked inhibition of most fulvestrant metabolic pathways by ketoconazole provided further evidence for the involvement of CYP3A4 in the microsomal metabolism of fulvestrant. Selective inhibitors of CYP 1A2, 2C9, 2C19 and 2D6 had no effect on fulvestrant metabolism. This was confirmed by the finding that recombinant CYP3A4 readily metabolises [^14^C]-fulvestrant, producing HPLC profiles that were qualitatively similar to human liver microsomal profiles. Metabolism was not observed in incubations of [^14^C]-fulvestrant with expressed CYP 1A2, 2C9, 2C19, 2D6, or flavin mono-oxygenase (FMO-3). Although fulvestrant readily undergoes CYP3A4-mediated metabolism when incubated with human liver microsomes, *in vitro* studies using human hepatocytes indicated that sulphate conjugation was a more predominant pathway. Therefore, CYP3A4 does not seem likely to have a major role in the overall clearance of the drug, and fulvestrant would not be expected to cause clinically significant drug interactions through inhibition of P450-mediated metabolism of co-administered agents.

Two randomised crossover studies have been conducted in healthy volunteers, to confirm that fulvestrant is not subject to CYP3A4 interactions that may potentially affect the safety or efficacy of the drug. These studies demonstrated that the pharmacokinetics of fulvestrant are not significantly affected by co-administration of compounds that induce (e.g. rifampicin) or inhibit (e.g. ketoconazole) CYP3A4 activity. In an additional randomised crossover study, fulvestrant did not significantly affect the pharmacokinetics of an agent (midazolam) that is a model substrate of CYP3A4 (Table 2

**Table 2:** Lack of potential for fulvestrant to be involved in significant CYP3A4-mediated drug interactions. Data are geometric least squares mean (glsmean) area under the concentration-time curve from 0 to time t (AUC_[0−t]_) for fulvestrant (rifampicin and ketoconazole studies) and AUC for midazolam (midazolam study)

**Fulvestrant 10 mg i.v.+rifampicin 600 mg (*n*=6)**	**Fulvestrant 10 mg i.v. alone (*n*=6)**	**Treatment effect^a^**	**Lower one-sided 95% CI**	**Upper 95% CI**
208 ng h ml^−1^	211 ng h ml^−1^	0.99^b^	0.65^b^	1.49
**Fulvestrant 8 mg i.v.+ketoconazole 400 mg (*n*=18)**	**Fulvestrant 8 mg i.v. alone (*n*=18)**	**Treatment effect^a^**	**Lower 90% CI**	**Upper 90% CI**
130.6 ng h ml^−1^	143.3 ng h ml^−1^	0.91	0.83	1.00
**Midazolam 7.5 mg+fulvestrant 36 mg i.m. (*n*=7)**	**Midazolam 7.5 mg alone (*n*=7)**	**Treatment effect^a^**	**Lower 90% CI**	**Upper 90% CI**
135 ng h ml^−1^	123 ng h ml^−1^	1.11	0.83	1.47

a Ratio of glsmeans;

b based on data from five volunteers.

) (Laight *et al*, 2003). These data reinforce the preclinical data and suggest that fulvestrant is unlikely to be the subject or cause of clinically significant drug interactions and no adjustments to the 250 mg i.m. dose are recommended when it is used in combination with agents that affect CYP3A4 activity. Further studies have shown that dosage adjustments are not required in patients with renal impairment or mild hepatic impairment (AstraZeneca, data on file).

## CONCLUSIONS

Fulvestrant is an ER antagonist that has a novel pharmacological profile and no agonist effects. Pharmacokinetic data have shown that i.m. injection of fulvestrant is the most effective mode of administration, with a single 250 mg i.m. injection of the long-acting fulvestrant formulation maintaining plasma concentrations within a 2–3-fold range above those predicted to be necessary for pharmacological activity over the dosing interval (28±3 days). Fulvestrant is highly metabolised and is mainly excreted in the faeces, and pharmacokinetic studies have also suggested that fulvestrant is unlikely to be the subject, or cause, of significant CYP3A4-mediated drug interactions.
